# Pharmacokinetic Interaction Study of Ketamine and Rhynchophylline in Rat Plasma by Ultra-Performance Liquid Chromatography Tandem Mass Spectrometry

**DOI:** 10.1155/2018/6562309

**Published:** 2018-05-23

**Authors:** Lianguo Chen, Weiwei You, Dingwen Chen, Yuan Cai, Xianqin Wang, Congcong Wen, Bo Wu

**Affiliations:** ^1^The Third Clinical Institute Affiliated to Wenzhou Medical University & Wenzhou People's Hospital, Wenzhou 325000, China; ^2^Analytical and Testing Center, School of Pharmaceutical Sciences, Wenzhou Medical University, Wenzhou 325035, China; ^3^Laboratory Animal Centre, Wenzhou Medical University, Wenzhou, China

## Abstract

Eighteen Sprague-Dawley rats were randomly divided into three groups: ketamine group, rhynchophylline group, and ketamine combined with rhynchophylline group (*n *= 6). The rats of two groups received a single intraperitoneal administration of 30 mg/kg ketamine and 30 mg/kg rhynchophylline, respectively, and the third group received combined intraperitoneal administration of 30 mg/kg ketamine and 30 mg/kg rhynchophylline together. After blood sampling at different time points and processing, the concentrations of ketamine and rhynchophylline in rat plasma were determined by the established ultra-performance liquid chromatography tandem mass spectrometry (UPLC-MS/MS) method. Chromatographic separation was achieved using a UPLC BEH C18 column (2.1 mm × 50 mm, 1.7 *μ*m) with carbamazepine as an internal standard (IS). The initial mobile phase consisted of acetonitrile and water (containing 0.1% formic acid) with gradient elution. Multiple reaction monitoring (MRM) modes of* m/z* 238.1 → 179.1 for ketamine,* m/z* 385.3 → 159.8 for rhynchophylline, and* m/z* 237.3 → 194.3 for carbamazepine (IS) were utilized to conduct quantitative analysis. Calibration curve of ketamine and rhynchophylline in rat plasma demonstrated good linearity in the range of 1-1000 ng/mL (r > 0.995), and the lower limit of quantification (LLOQ) was 1 ng/mL. Moreover, the intra- and interday precision relative standard deviation (RSD) of ketamine and rhynchophylline were less than 11% and 14%, respectively. This sensitive, rapid, and selective UPLC-MS/MS method was successfully applied to pharmacokinetic interaction study of ketamine and rhynchophylline after intraperitoneal administration. The results showed that there may be a reciprocal inhibition between ketamine and rhynchophylline.

## 1. Introduction

Ketamine is clinically used for surgical anesthesia [1, 2]. Commonly known as “K powder”, ketamine was epidemically abused in the United States in the early 70s of last century [3, 4]. With the widespread abuse of “party drug” worldwide since the 1990s, the abuse of ketamine quickly spread to the Asian region or even mainland China [5, 6]. Ketamine was often abused in entertainment venues, making it one of the relatively popular new drugs currently [7, 8].

Traditional Chinese medicines such as* Gastrodia Elata *and* Uncaria* which can suppress hyperactive liver for calming endogenous wind, together with drugs which can warm kidney and activate yang and disperse stagnated liver qi for relieving qi stagnation, are used in clinical treatment with a good therapeutic effect [9, 10]. Therefore, as a common traditional Chinese medicine in modern detoxification compound,* Uncaria* is frequently used as the main drug in detoxification preparations, such as Kangfuxin capsule, composite Dongyuan Gao, Shutongan capsule, and other preparations. Clinical studies have confirmed that these traditional Chinese medicine compounds are effective in controlling the withdrawal symptoms, relieving mental dependence, and reducing relapse [11, 12]. Moreover, they are safe and effective, with no obvious side effects.

Consequently, in the present study, we established a UPLC-MS/MS method for the quantification of ketamine and rhynchophylline in rat plasma. Meanwhile, we investigated the pharmacokinetic interaction of them to explore the anti-ketamine addiction mechanism of rhynchophylline.

## 2. Materials and Methods

### 2.1. Chemicals and Reagents

**Figure 1:**
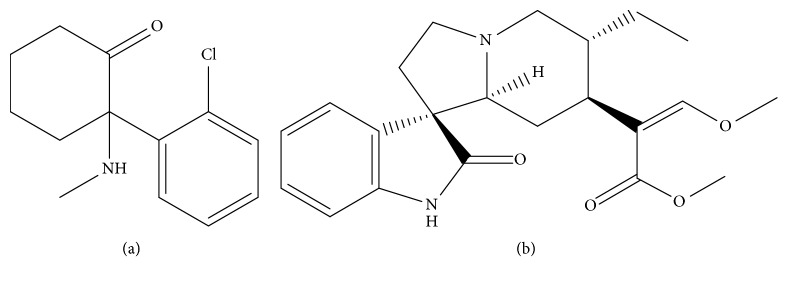
Molecular structure of ketamine (a) and rhynchophylline (b).

Ketamine (purity > 98%, Figure 1(a)), rhynchophylline (purity > 98%, Figure 1(b)), and carbamazepine (IS, purity > 98%) were purchased from Chinese Biopharmaceutical Institute (Beijing, China). High performance liquid chromatography-grade acetonitrile and methanol were purchased from Merck Company (Darmstadt, Germany). Ultra-pure water was prepared by a Milli-Q purification system (Millipore Bedford, MA). Sprague-Dawley rats (200-220 g) were purchased from Laboratory Animal Centre of Wenzhou Medical University [2].

### 2.2. Instrumentation and Conditions

A UPLC-MS/MS system with ACQUITY I-Class UPLC and a XEVO TQS-micro triple quadrupole mass spectrometer (Waters Corp., Milford, MA, USA) and Masslynx 4.1 software (Waters Corp.) was used for data acquisition and instrument control. Chromatographic separation was achieved using a UPLC BEH C18 column (2.1 mm × 50 mm, 1.7 *μ*m) (Waters Corp., Milford, MA, USA) maintained at 40°C. The temperature of UPLC-MS sampling chamber was maintained at 10°C. The initial mobile phase consisted of acetonitrile and water (containing 0.1% formic acid) with gradient elution at a flow rate of 0.4 mL/min. Elution was in a linear gradient, where the acetonitrile content was maintained at 10% between 0 and 0.2 min, increased to 80% between 0.2 and 1.5 min, maintained at 80% between 1.5 and 2.0 min, then decreased to 10% between 2.0 and 2.5 min, and maintained at 10% between 2.5 and 4.0 min. The total run time of the analytes was 4 min.

**Figure 2:**
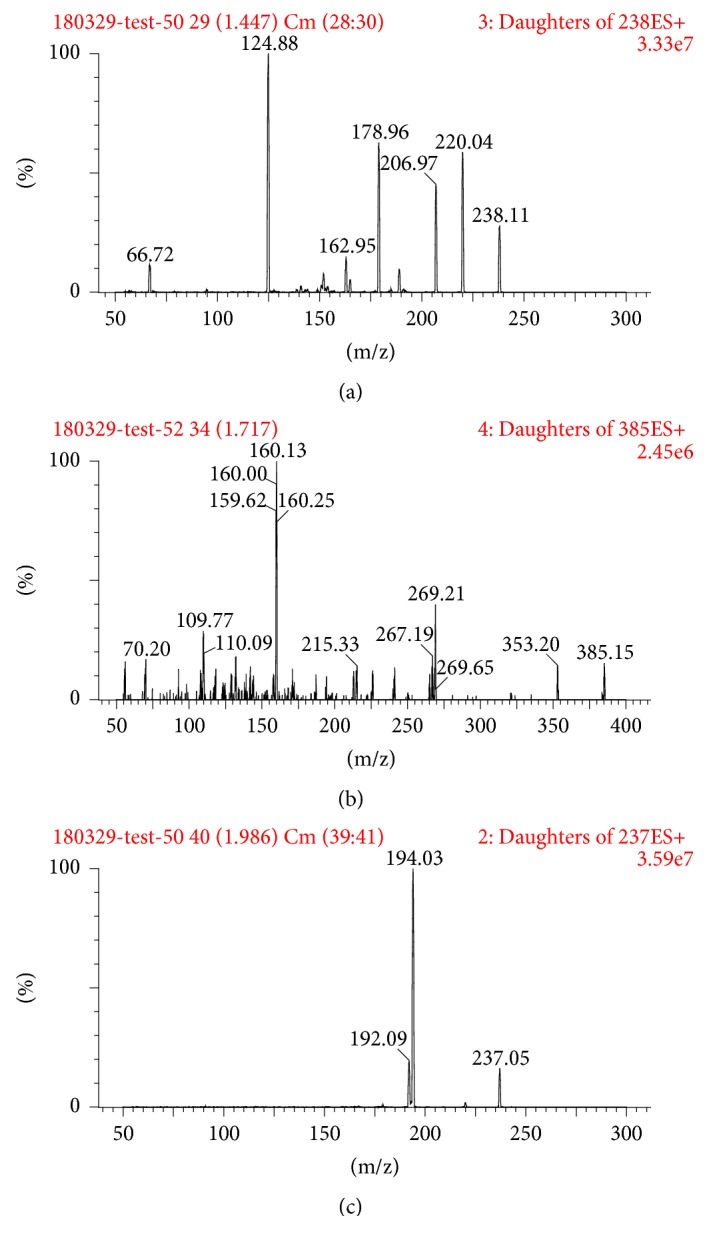
Mass spectra of ketamine (a), rhynchophylline, (b) and carbamazepine (IS, c).

Nitrogen was used as the desolvation gas (1000 L/h) and cone gas (50 L/h). Ion monitoring conditions were defined as capillary voltage of 1.5 kV, source temperature of 150°C, and desolvation temperature of 500°C [13]. MRM modes of m/z 238.1 → 179.0 for ketamine, m/z 385.2 → 160.1 for rhynchophylline, and m/z 237.1 → 194.0 for carbamazepine (IS) were utilized to conduct quantitative analysis, Figure 2.

### 2.3. Quality Control Samples Preparation

The stock solutions of ketamine (1.0 mg/mL), rhynchophylline (1.0 mg/mL), and carbamazepine (IS) were prepared in methanol-water. The working standard solution of IS was prepared from the IS stock solution by dilution with methanol; working solutions for calibration and controls were prepared from stock solutions similarly, using methanol diluent. All of the solutions were stored at 4°C and were brought to room temperature before use [14].

### 2.4. Calibration Standards Preparation

Ketamine and rhynchophylline calibration standards were prepared by spiking blank rat plasma with appropriate amounts of the working solutions [15]. Calibration plots were offset to range between 1 and 1000 ng/mL for ketamine or rhynchophylline in rat plasma at 1, 5, 20, 50, 100, 200, 500, and 1000 ng/mL. Quality control (QC) samples were prepared in the same manner as the calibration standards, in three different plasma concentration levels (2, 90, and 900 ng/mL).

### 2.5. Sample Preparation

One hundred microliter of plasma sample was mixed with 200 *μ*L of acetonitrile containing 50 ng/mL carbamazepine (IS) in a 1.5-mL centrifuge tube and then extracted by vortexing for 1.0 min to deproteinize the endogenous protein. After centrifugation at 14900* g *for 10 min at 4°C, 100 *μ*L of the supernatant was collected into the inner lining-pipe of a sample vial. Two microliters of the supernatant was injected into the UPLC-MS/MS system for analysis.

### 2.6. Pharmacokinetic Study

Eighteen Sprague-Dawley rats (200-220 g) were randomly divided into three groups: ketamine group, rhynchophylline group, and ketamine combined with rhynchophylline group (*n *= 6) [6]. Ketamine and rhynchophylline were dissolved in 2% dimethyl sulfoxide (DMSO). Two groups received a single intraperitoneal (ip) administration (30 mg/kg ketamine and 30 mg/kg rhynchophylline, respectively), and the third group received combined intraperitoneal (ip) administration of 30 mg/kg ketamine and 30 mg/kg rhynchophylline together. Blood samples (0.3 mL) from the tail vein were collected into 1.5 mL heparinized polypropylene tubes at 0.25, 1, 3, 6, 8, 10, and 24 h after intraperitoneal administration. The samples were immediately centrifuged at 13000 rpm for 10 min at 4°C. Then the plasma was transferred to a new 1.5 mL tube and stored at −20°C until analysis.

Plasma concentration versus time data for each rat was analyzed by DAS (Drug and Statistics) software (version 2.0, China Pharmaceutical University). The area under the plasma concentration-time curve (AUC), mean residence time (MRT), plasma clearance (CL), apparent volume of distribution (V), maximum plasma concentration (C_max_), and half-life (t_1/2_) were estimated using noncompartmental calculations performed with DAS software [15].

## 3. Results

### 3.1. UPLC-MS/MS Method Verification

**Figure 3:**
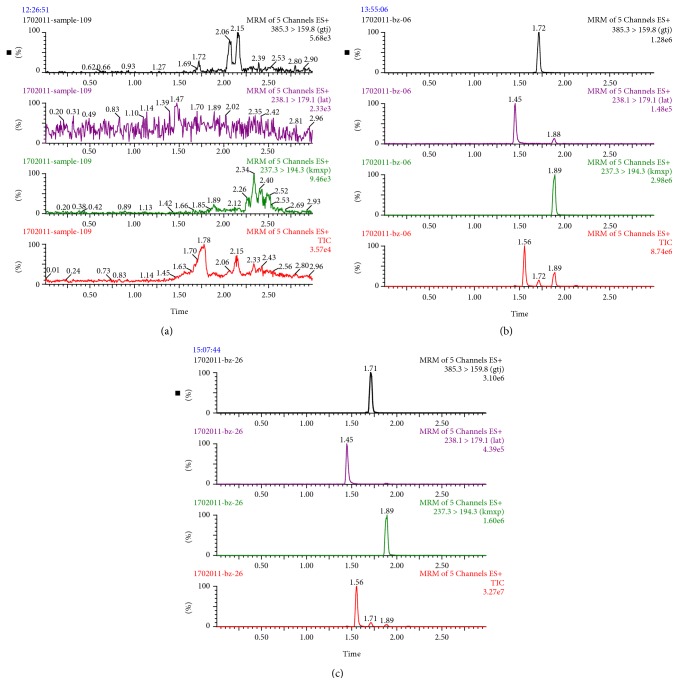
Representative UPLC-MS/MS chromatograms: (a) blank plasma; (b) blank plasma spiked with ketamine, rhynchophylline, and carbamazepine (IS); (c) a rat plasma sample 2 h after intraperitoneal administration.

Typical UPLC-MS/MS chromatograms of blank plasma, blank plasma spiked with ketamine, rhynchophylline, and carbamazepine (IS), and plasma samples collected from the caudal vein of rats were shown in Figure 3. No interference of visible impurity and endogenous substances was observed, indicating that the analyte of interest and IS were efficiently separated by the optimized gradient elution procedure.

Calibration curve of ketamine and rhynchophylline in rat plasma demonstrated good linearity in the range of 1-1000 ng/mL. Typical regression equations were as follows: Y_1_ = 0.00041X_1_ + 0.00094, r = 0.9996; Y_2_ = 0.00454X_2_ - 0.00928, r = 0.9975. Y_1_ represents the ratios of peak intensity of ketamine to the internal standard, and X_1_ represents the concentration of ketamine in plasma; Y_2_ represents the ratios of peak intensity of rhynchophylline to the internal standard, and X_2_ represents the concentration of rhynchophylline in plasma. The LLOQ of ketamine and rhynchophylline in rat plasma was 1 ng/mL.

As shown in Table 1, the intra- and interday precision RSD at three concentration levels of ketamine were all less than 11% and the accuracy was in the range of 94.3% to 111.8%. The mean recovery was higher than 72.6% and the matrix effect was between 103.8% and 108.7%. It is known from Table 2 that the intra- and interday precision RSD of rhynchophylline were less than 14% and the accuracy was in the range of 93.3% to 110.2%. The mean recovery was higher than 66.2% and the matrix effect was between 98.8% and 102.7%. These results demonstrated that the precision, accuracy, recovery, and matrix effect of the established UPLC-MS/MS method were all conformed to the pharmacokinetic requirements of ketamine and rhynchophylline.

### 3.2. Pharmacokinetic Study

**Figure 4:**
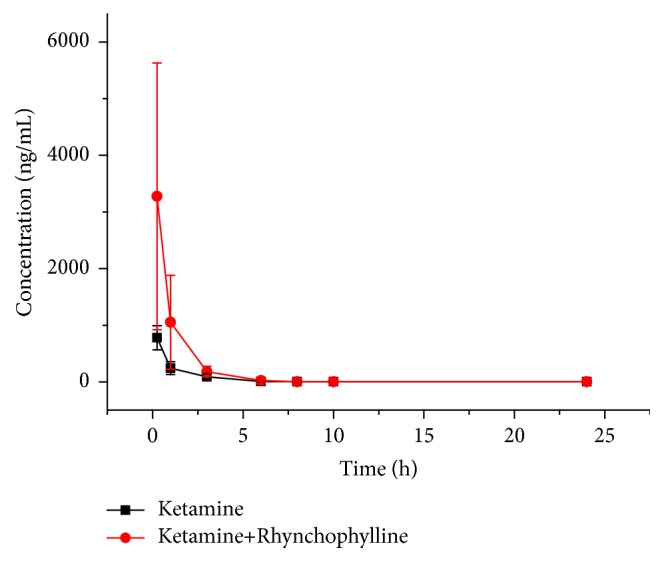
Mean plasma concentration-time curves of ketamine after intraperitoneal administration of a single 30 mg/kg ketamine and 30 mg/kg ketamine combined with 30 mg/kg rhynchophylline in rats.

**Figure 5:**
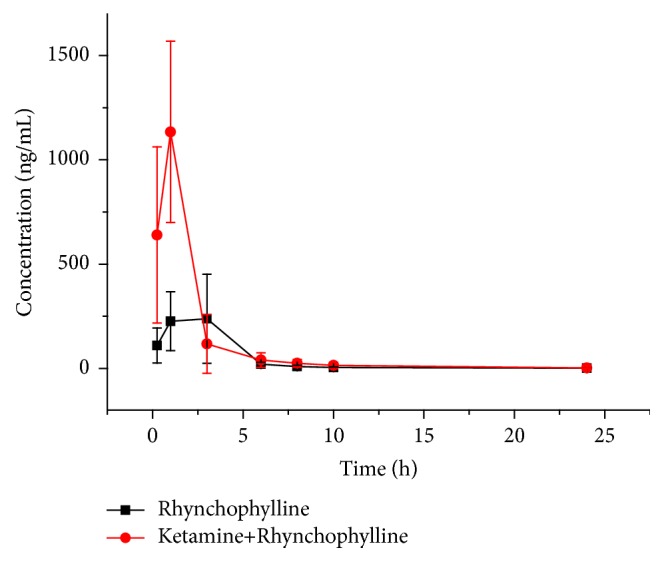
Mean plasma concentration-time curves of rhynchophylline after intraperitoneal administration of a single 30 mg/kg rhynchophylline and 30 mg/kg ketamine combined with 30 mg/kg rhynchophylline in rats.

The mean plasma concentration-time curve of ketamine is shown in Figure 4; the mean plasma concentration-time curve of rhynchophylline is shown in Figure 5; primary pharmacokinetic parameters which are based on noncompartmental model analysis are summarized in Tables 3 and 4.

## 4. Discussion

In the present study, a sensitive, rapid, and selective UPLC-MS/MS method for the quantitation of ketamine and rhynchophylline in rat plasma was established, utilizing 100 *μ*L of plasma with an LLOQ of 1 ng/mL and 4 min total run time. The UPLC-MS/MS method was successfully applied to pharmacokinetic interaction study of ketamine and rhynchophylline, which suggested that there may be a reciprocal inhibition between them.

The combination of various drugs is very common in clinical treatment and the interaction of drugs has drawn more and more attention. The drug metabolic interaction generated by the induction or inhibition of Chinese herbal medicine on hepatic drug-metabolizing enzymes (CYP450) is an important part and the most common cause of the interaction between Chinese and Western medicines [16]. Rhynchophylline and ketamine are both noncompetitive antagonists of N-methyl-D-aspartic acid receptors (NMDA) and can be used to interfere with ketamine addiction when they are used together [17]. Therefore, it is of great significance to study the pharmacokinetic changes after the combined use of rhynchophylline and ketamine. At present, no relevant reports have been found yet.

Most interactions of herbal medicine and Western medicine mainly appeared by affecting the emergence of cytochrome P450, UDP-glucuronosyl transferase (UGT), or drug transport protein [18, 19]. Herbal medicine can inhibit or induce these enzymes or protein to increase or decrease the medical concentration in blood, viscera, urine, or bile and proceed to cause the change of pharmacokinetic parameters and lead to ineffective treatment of the medicine or potential adverse reactions.

The results showed that primary pharmacokinetic parameter of ketamine such as C_max_ was 779.5 ± 212.6 ng/mL, AUC_(0-*∞*)_ was 1083.4 ± 158.7 ng/mL*∗*h, MRT_(0-*∞*)_ was 1.4 ± 0.3 h, CL was 28.1 ± 3.7 L/h/kg, t_1/2_ was 5.4 ± 2.6 h, and V was 208.1 ± 84.3 L/kg. Compared with single ketamine group, there were significant differences of C_max_, AUC, V, and CL_z/F_ after administration of ketamine combined with rhynchophylline. C_max_ and AUC were increased 3.2 and 2.8 times, respectively, after combined use, suggesting that rhynchophylline may increase the absorption of ketamine, while V, CL, and t_1/2_ were only 17.0%, 37.0%, and 44.4%, respectively, of those of single ketamine group, suggesting that the distribution and metabolism of ketamine were obviously decreased after being combined with rhynchophylline. The investigation of Hijazi Y et al. showed that the subtypes of CYP 450 (CYP2C9, CYP2B, and CYP3A) participated in N-demethylation of ketamine in rats liver, and ketamine may interact with the aforesaid enzyme substrate* in vivo *[20]. Therefore, rhynchophylline may theoretically induce CYP2B6, CYP3A4, and CYP2C9 to accelerate the metabolism of ketamine. However, it was inconsistent with our results, indicating that rhynchophylline mainly inhibited ketamine metabolism through other ways. The obvious medical interaction appeared by regulation of medical absorption, distribution, metabolism, and excretion with CYP450 as well as UDP-glucuronosyl transferase (UGT) and drug transport protein (OCT1, OCT2, OAT1, OAT3, OATP1B1, OATP1B3, P-gp, and BCRP) [19]. The binding rate of drug plasma protein was also the important factor to the interaction, especially to the approachable drug with plasma protein [21]. In addition, the constituent of* Uncaria* was complex; there were isorhynchophylline, corynoxeine, isocorynoxeine, corynantheine, corynoxein, isocorynoxeine, and so forth besides rhynchophylline [22, 23]. Therefore, the intervening results of* Uncaria* decoction on hepatic enzyme were not able to directly explain the effect of rhynchophylline on hepatic enzyme. There were no relevant reports on these influence factors, and further verification was needed on the interaction of rhynchophylline and ketamine. At present, although the relevant mechanism was not definite, attention should be given on the effect of increasing absorption and inhibiting metabolism of ketamine by rhynchophylline, because it may result in an unnecessary liver injury or other adverse reactions if the plasma concentration of ketamine was too high [24].

The results also showed that primary pharmacokinetic parameter of rhynchophylline such as C_max_ was 314.9 ± 162.1 ng/mL, AUC_(0-*∞*)_ was 1094.8 ± 715.1 ng/mL*∗*h, MRT_(0-*∞*)_ was 2.9 ± 0.8 h, CL was 40.9 ± 29.0 L/h/kg, t_1/2_ was 2.9 ± 2.2 h, and V was 146.3 ± 100.5 L/kg. Compared with single rhynchophylline group, there were significant differences of C_max_, AUC, and CL after administration of ketamine combined with rhynchophylline. C_max_ and AUC were increased 2.6 and 1.4 times, respectively, after combined use, suggesting that ketamine may increase the absorption of rhynchophylline, while CL was decreased by 67.4% and t_1/2_ was extended by 3.0 times (no significant differences), suggesting that ketamine may induce the metabolism of rhynchophylline. Ketamine had always been considered as a hepatic enzyme inducer previously, but other research* in vivo* and* in vitro* showed that ketamine can inhibit the activity of CYP1A, CYP2A, CYP2B, CYP2C, CYP2D1, and CYP3A [24, 25]. The result of this contradiction was probably caused by different time and frequency of administration. Repeated administration and time of ketamine were required to gain the induction effect on hepatic enzyme. However, rhynchophylline was mainly metabolized through the hydroxylation of CYP2D, CYP1A1/2, and CYP2C [26]. Therefore, single administration of ketamine may decrease the activity of CYP2D, CYP1A1/2, and CYP2C to inhibit the hydroxylation of rhynchophylline. This hypothesis is consistent with our result, and further elaboration was needed for the detailed reasons.

## 5. Conclusion

In the present study, we investigated the pharmacokinetic interaction of ketamine and rhynchophylline based on a newly established UPLC-MS/MS method, which showed that rhynchophylline can increase the absorption and inhibit the metabolism of ketamine; meanwhile ketamine can increase the absorption and induce the metabolism of rhynchophylline. Hepatic enzyme was not able to completely explain their interaction* in vivo* of rats, and there were no relevant reports of UDP-glucuronosyl transferase, drug transport protein, and binding rate of plasma protein on this result. Further researches still need to be conducted to study the relevant mechanism, and attention should be given on the results that may be caused by their interactions clinically.

## Figures and Tables

**Table 1:** Precision, accuracy, and recovery of ketamine in rat plasma (*n* = 6).

Concentration (ng/mL)	Precision (RSD%)		Accuracy (%)		Recovery (%)	Matrix effect (%)
	Intraday	Interday	Intraday	Interday		
2	10.0	10.4	99.1	95.4	72.6	108.7
90	7.9	5.6	94.3	111.8	73.3	103.8
900	6.0	7.9	108.7	94.8	74.9	104.3

**Table 2:** Precision, accuracy, and recovery of rhynchophylline in rat plasma (*n* = 6).

Concentration (ng/mL)	Precision (RSD%)		Accuracy (%)		Recovery (%)	Matrix effect (%)
	Intraday	Interday	Intraday	Interday		
2	12.5	13.3	110.2	90.4	68.0	102.7
180	9.7	7.9	93.3	103.6	66.2	100.1
900	4.7	11.2	100.4	104.2	69.9	98.8

**Table 3:** Primary pharmacokinetic parameters after intraperitoneal administration of ketamine in rats (*n* = 6).

Parameters	Unit	Ketamine	Ketamine + rhynchophylline
AUC_(0-t)_	ng/mL*∗*h	1080.4± 156.6	4085.7 ± 2784.8^*∗*^
AUC_(0-*∞*)_	ng/mL*∗*h	1083.4 ± 158.7	4086.7 ± 2784.3^*∗*^
MRT_(0-t)_	h	1.4 ± 0.3	1.0 ± 0.3
MRT_(0-*∞*)_	h	1.4 ± 0.3	1.1 ± 0.3
t_1/2_	h	5.4 ± 2.6	2.4 ± 1.5
CL	L/h/kg	28.1 ± 3.7	10.4 ± 5.6^*∗∗*^
V	L/kg	208.1 ± 84.3	35.2 ± 28.3^*∗∗*^
C_max_	ng/mL	779.5 ± 212.6	3275.7 ± 2357.1^*∗*^

Compared to ketamine group, ^*∗*^*P* < 0.05, ^*∗∗*^*P* < 0.01.

**Table 4:** Primary pharmacokinetic parameters after intraperitoneal administration of rhynchophylline in rats (*n* = 6).

Parameters	Unit	Rhynchophylline	Ketamine + rhynchophylline
AUC_(0-t)_	ng/mL*∗*h	1090.9 ± 710.8	2620.0 ± 1291.2^*∗*^
AUC_(0-*∞*)_	ng/mL*∗*h	1094.8 ± 715.1	2650.3 ± 1269.4^*∗*^
MRT_(0-t)_	h	2.9 ± 0.7	1.8 ± 0.5^*∗*^
MRT_(0-*∞*)_	h	2.9 ± 0.8	3.1 ± 2.6
t_1/2_	h	2.9 ± 2.2	11.5 ± 19.1
CL	L/h/kg	40.9 ± 29.0	13.3 ± 5.1^*∗*^
V	L/kg	146.3 ± 100.5	264.0 ± 473.0
C_max_	ng/mL	314.9 ± 162.1	1133.7 ± 433.9^*∗∗*^

Compared to ketamine group, ^*∗*^*P* < 0.05, ^*∗∗*^*P* < 0.01.

## Data Availability

The data used to support the findings of this study are available from the corresponding author upon request.
